# Lipidomic analysis of isolated lipid droplets reveals potential metabolic pathway differences between medulloblastoma subgroups

**DOI:** 10.3389/fnins.2026.1863712

**Published:** 2026-09-01

**Authors:** Kian Cotton, Jack Stafford, Christopher Edwards, Maria Victoria Niklison-Chirou

**Affiliations:** 1 Department of Life Sciences, University of Bath, Bath, United Kingdom; 2 Blizard Institute, Barts and The London School of Medicine and Dentistry, Queen Mary University of London, London, United Kingdom

**Keywords:** cancer, lipid droplet, lipidomic, medulloblastoma, metabolism

## Abstract

**Introduction:**

Medulloblastomas (MBs) are a highly aggressive paediatric brain tumour which is very difficult to treat. Lipid metabolism has emerged as a crucial determinant of tumour progression, metastasis and therapy resistance in MBs. Lipid droplets (LDs) are lipid-rich organelles that store neutral lipids; their biology and function remain poorly characterised within MBs.

**Methods:**

MB cells from the low-aggressive Sonic hedgehog (SHH) subgroup (DAOY, UW228-2) and the high-aggressive group 3 (G4) and group 4 (G4) subgroups (D458, D283) were treated with 80 μM oleic acid (OA), 50 μM cholesterol (Chol) or a mix to induce LD formation. LDs were isolated, and lipidomic analysis was performed.

**Results:**

No lipid species were consistently upregulated across all cell lines under any treatment condition. SHH cell lines exhibited a broad and consistent lipidomic response across all three treatments, resulting in a pronounced triacylglyceride (TAG) and cholesterol ester (CE) storage profile. The more aggressive subgroups exhibited a more selective, OA-driven phenotype, characterised by longer-chain TAG and CE species. Across all cell lines, OA produced the strongest LD expansion and metabolic separation, whereas Chol elicited a more restricted but highly subgroup-specific signature. G3/G4 Chol responses provide evidence for lipid metabolism rewiring, which could be responsible for the more aggressive phenotype.

**Discussion:**

These findings demonstrate that MB subtypes not only exhibit distinct basal lipid signatures but also engage in fundamentally different lipid remodelling in response to FA overload. These distinct lipid-handling signatures display metabolic heterogeneity across MB subgroups and suggest subtype-specific vulnerabilities. Overall, these findings position LD remodelling as a potential metabolic target of MB cells.

## Introduction

Medulloblastomas (MB) are the most common malignant paediatric brain tumours, originating in the cerebellum, and accounting for 10–15% of paediatric brain cancers (Lee et al., 2022). Genomic studies reveal four primary molecular subgroups: wingless (WNT - good prognosis), sonic hedgehog (SHH - poor/intermediate prognosis), Group 3 (G3 – poor prognosis), and Group 4 (G4 - intermediate prognosis) with distinct genetic drivers, clinical outcomes and patterns of metastasis (Northcott et al., 2011; Huang et al., 2024). Despite subgroup classification studies and advances in multimodal therapies, patients still face poor prognoses and/or significant long-term side effects, highlighting the unmet clinical need.

Cancer cells undergo significant metabolic changes to support proliferation. In recent studies, lipid metabolism has emerged as a critical pathway that supports tumour development (Martin-Perez et al., 2022). Lipids are essential for membrane structure, energy storage, signalling and regulation of oxidative stress (Fernández-García et al., 2023). However, an overaccumulation of lipids can lead to induction of apoptosis, and so storage capabilities are required.

**Figure 1:**
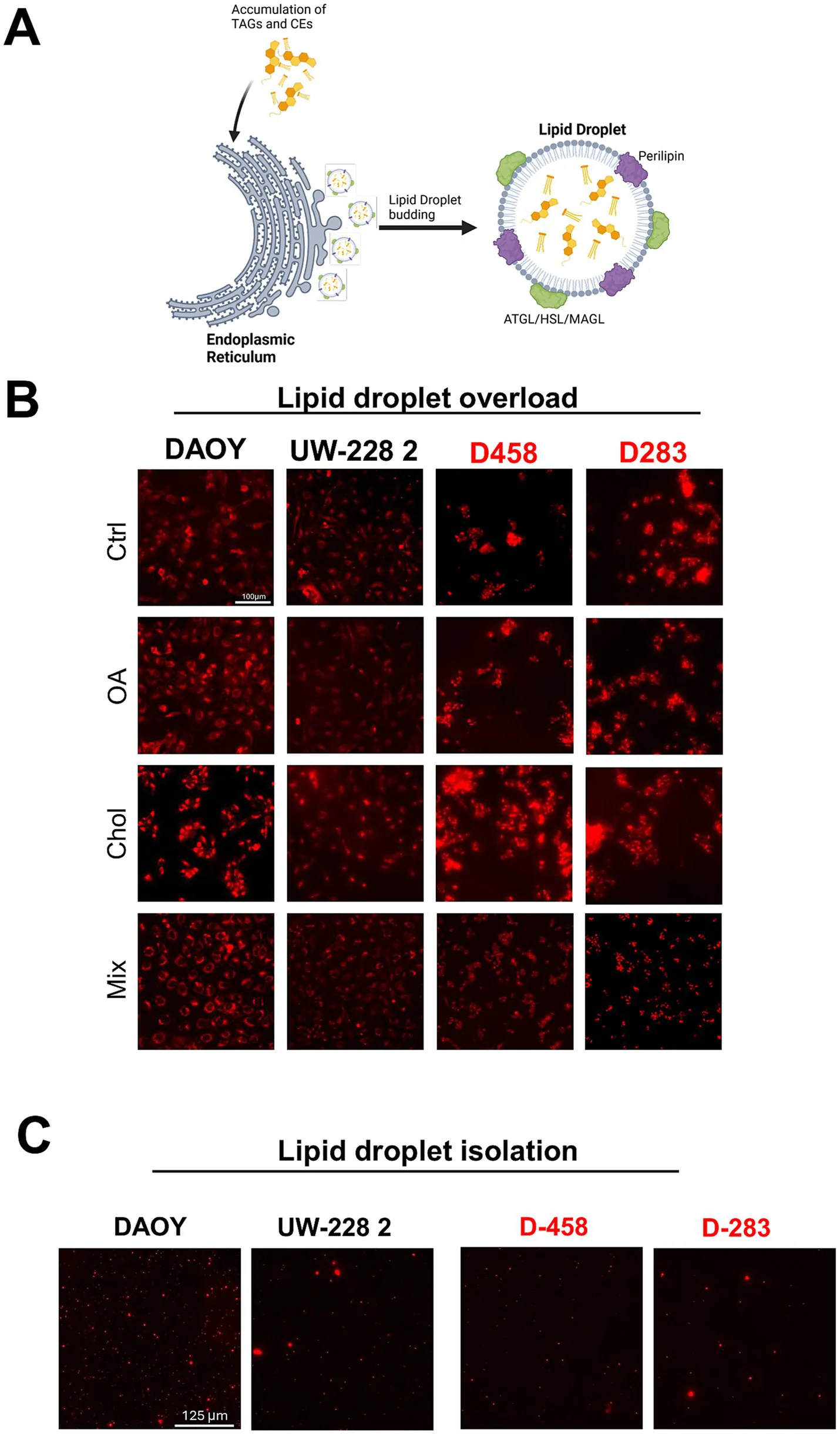
Lipid droplets are successfully isolated from all four MB cell lines. **(A)** Schematic representation of LD architecture and composition. Droplets are composed of a phospholipid monolayer encasing a hydrophobic neutral lipid core dominated by TAGs, CEs and DAGs. The outer membrane is embedded with several functional and structural proteins such as the perilipins (PLIN1-5), adipose triglyceride lipase (ATGL) and hormone-sensitive lipase (HSL). Schematic of Lipid droplet composition and structure” created in BioRender. Cotton, K. is licensed under CC BY 4.0. **(B)** Representative fluorescent imaging of DAOY, UW228-2, D458 and D283 cells stained with Nile Red (Red) at rest or following treatment with 80 μM OA, 50 μM Chol or a mix. Taken at 20x magnification, scale bar = 100 μm. **(C)** Validation of lipid droplets successfully isolated from DAOY, UW228-2, D458 and D283 MB cells following treatment with 80 μM OA. Samples were stained with Nile Red (Red) and confirmed using fluorescence microscopy. Scale bar = 125 μm.

Lipid droplets (LDs) are complex organelles found within the cytoplasm of most mammalian cells; they are now understood to perform diverse functions beyond simple energy storage (Figure 1A) (Zadoorian et al., 2023). They consist of a neutral lipid core composed primarily of triacylglycerols (TAGs) and cholesterol esters (CEs). LDs are encased by a phospholipid monolayer derived from the endoplasmic reticulum, coated by characteristic structural and functional proteins, including members of the perilipin family (PLIN1-5), which regulate LD stability, expansion and lipase accessibility or enzymes involved in lipolysis, such as adipose triglyceride lipase (ATGL/PNPLA2) and hormone-sensitive lipase (HSL) (Figure 1A) (Shaw et al., 2013).

Lipids can be metabolised through β-oxidation in the mitochondria to produce energy. It has been shown that LDs and mitochondria physically interact, facilitating direct lipid transfer, which is essential for cellular metabolism (Wang et al., 2025; Talari et al., 2023). Most importantly, LD accumulation provides a protective mechanism against lipotoxicity (Bingi et al., 2024). Elevated LD formation would therefore be beneficial to tumour cells, to support rapid proliferation while avoiding lipotoxicity.

A recent plasma lipidomic study in MB patients identified 97 lipid species that were differentially expressed between patients and controls. The most significant changes were observed in glycerophospholipid, sphingolipid, and the linoleic acid metabolism pathways (Shi et al., 2025).

Identifying lipid-metabolic dependencies (for instance, a subgroup enriched in CEs via Sterol O-Acetyltransferase 1 (SOAT1) activity) may unmask novel vulnerabilities, providing a less toxic, precise intervention.

In this study, we applied high-resolution untargeted lipidomic analysis to LDs isolated from SHH and G3/G4MB subgroups, focusing on identifying subgroup-specific differences in storage and signalling lipids, including TAGs, TGs and CEs. With the aim of discovering potential metabolic rewiring in MB cell lines and subgroups.

## Materials and methods

### Cell culture

Four human MBs cell lines were used: DAOY, UW228-2, D458, and D283. D458 (G3) cells were kindly gifted by Dr. Silvia Marino, Queen Mary University of London. DAOY (SHH), UW228-2 (SHH) and D283 (G3/G4) were purchased from ATCC.

For standard culture conditions, DAOY, UW228-2, and D458 were grown in GIBCO™ Dulbecco’s Modified Eagle Medium (DMEM), high glucose, GlutaMAX™ composition (ThermoFisher, #31966021) supplemented with 10% foetal bovine serum (FBS) (GIBCO™, ThermoFisher #10270106), 1% penicillin–streptomycin (P/S) (Merck, #P0781) and 1% non-essential amino acids (NEAA) (Merck, #M7145), referred to as ‘complete media’. D283 cells were cultured separately in GIBCO™ RPMI 1640 Medium (ThermoFisher, #11875085) supplemented with 10% FBS, 1% P/S and 1% NEAA, hereafter termed ‘complete RPMI’. All cell lines were incubated at 37 °C with 5% CO₂. Cell lines were sub-cultured when they reached approximately 70–80% confluency.

### Immunofluorescence

Coverslips were sterilised with 100% ethanol (EtOH) before being set up in 12-well plates (ThermoFisher, #10098870), air-dried, and then laid flat. To enhance attachment of semi-suspension cell lines, coverslips were sequentially coated with poly-L-ornithine (Merck, #P3655) for 1 h, followed by overnight treatment with laminin (R&D Systems, USA #3432–010-01). Seeding was performed to achieve 50% confluent cover slips in complete DMEM (DAOY, UW228-2, D458) or complete RPMI (D283).

Oleic acid (OA) (Merck, #O1257) was obtained in water-soluble form and dissolved in sterile dH_2_O. Cholesterol (Chol) (Merck, #C3045) was dissolved in ethanol. Cells were seeded at the required density and treated with 80 μM OA, 50 μM Chol, or a combination (Mix) overnight.

Following treatment, cells were fixed in 4% paraformaldehyde solution for 15 min and stained with DAPI (1:5000) (MedChemExpress, #HY-D0814) and Nile Red (1:300) (Merck, #N3013), protected from light. DAPI was used for nuclei visualisation and Nile Red to stain neutral lipids. Cover slips were mounted onto microscope slides with Fluoromount-G™ (Invitrogen, #00–4,958-02) and sealed using nail varnish. An EVOS™ M5000 microscope (Invitrogen, #AMF5000) was used for imaging at 20X magnification.

### Lipid droplet isolation

LD isolation was performed using an Abcam isolation kit (Abcam, #Ab242290) according to the manufacturer’s instructions. Briefly, DAOY, UW228-2, D458, and D283 cells were plated at 2 × 10^6^ cells in 12 mL. Cells were stimulated to induce LDs, as previously described, after reaching 80% confluency. Each treatment condition (80 μM OA, 50 μM Chol, or a mixture) was performed in triplicate with two dishes per replicate to ensure an adequate number of lipid droplets could be isolated. Cells were incubated overnight, detached with trypsin, and pelleted at 1000xg for 5 min. The supernatant was removed and cells washed with 10 mL of phosphate buffered saline (PBS). The following solution was centrifuged again at 1000xg for 5 min, the supernatant was removed, and the cells were resuspended in 1 mL of PBS. Cells were then centrifuged again at 1000xg for 5 min, PBS was removed, and the pellet was resuspended in 200 μL of Reagent A. This was then incubated on ice for 10 min. Following this, 800 μL of Reagent B was added and incubated for a further 10 min on ice. Cells were then passed through a 3/4-inch 27-gauge needle five times to homogenise the remaining cell count. The homogenate was centrifuged at 100xg for 5 s, and 600uL of Reagent B was layered on top by dropwise addition. The final mixture was then centrifuged again for an additional 3 h at 20,000xg at 4 °C. A 270 μL fraction containing LDs was carefully collected from the upper layer of the homogenate and transferred to a clean microcentrifuge tube.

To verify successful lipid droplet isolation, a 5 μL aliquot of the sample was stained with Nile Red and examined using the EVOS™ M5000. Following confirmation, the isolated LDs were stored at −80 °C until further analysis.

### Lipidomic profiling

Lipidomic analysis was performed by the FGCZ metabolic team at the University of Zurich using an omics-scale, high-throughput quantitative LC–MS/MS (Liquid Chromatography–Tandem Mass Spectrometry) platform for circulatory lipid phenotyping as previously described (Medina et al., 2023).

### Statistical analysis

Statistical analysis of peak intensities and figure generation were carried out using MetaboAnalyst 6.0^1^. The dataset was uploaded in an unpaired column format, transformed by Log (base10), and auto scaled before analysis. Principal Component Analysis (PCA) plots were used to examine differences between treatment groups. Volcano plots were generated to identify lipid species significantly altered between treatment and control. Significance thresholds were set at a more lenient than usual value of fold-change>2 and *p* < 0.1 allowing for wider analysis of the dataset whilst still combining two parameters. One-way ANOVA was performed to identify cell-specific differences (FDR *p* < 0.1), followed by a *post hoc* Tukey’s test to determine pairwise significance (*p* < 0.05).

## Results

### MB subgroups show distinct clustering between SHH and G3/G4

Before characterising lipid composition, the successful induction and isolation of LDs from each cell line was confirmed. Nile Red labelled LDs were detected in all preparations following OA, Chol or mix overload (Figure 1B) and after isolation (Figure 1C), indicating enrichment of LDs suitable for lipidomic analysis.

Under control conditions, DAOY, and UW228-2 cells showed low basal LD content, whereas D458 and D283 exhibited modest staining when compared to cells treated with lipid overload (Figure 1B; Supplementary Figure S1–S4).

Principal component analysis (PCA) was performed to provide an initial, global overview of the metabolic landscape. All additional PCA statistics are provided in Supplementary Table 1.

**Figure 2:**
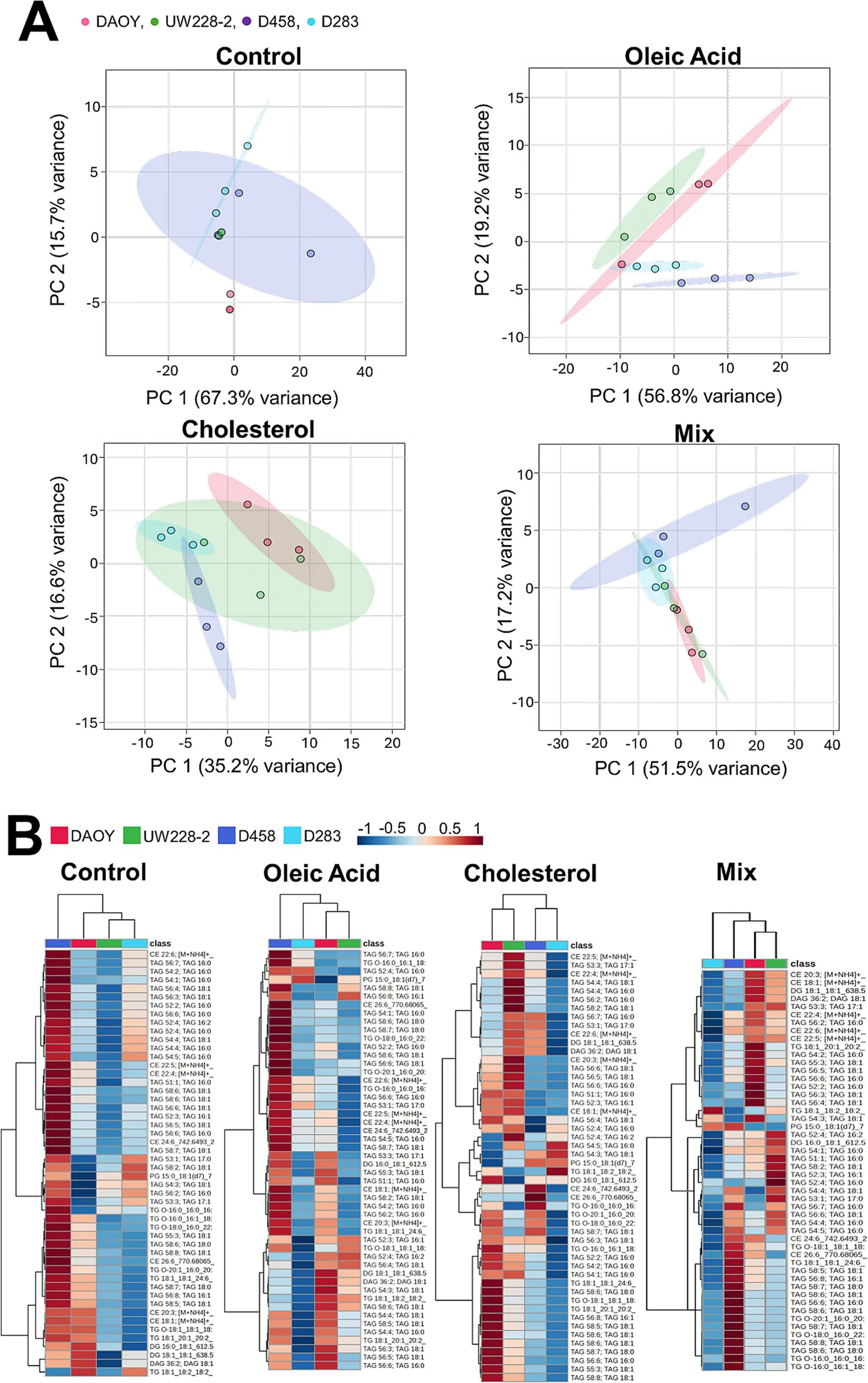
Oleic acid and cholesterol treatment induce LD formation in medulloblastoma cells. **(A)** Principal component analysis of lipid droplets isolated from DAOY, UW228-2, D458 or D283 cells, untreated (top left), or treated with OA (top right), Chol (bottom left) or mix (bottom right). Individual points represent independent biological replicates, separated by colour into cell lines, DAOY (red), UW-228-2 (green), D458 (purple) and D283 (light blue). **(B)** Heat maps of detected lipid species recorded during analysis of lipid droplets isolated from DAOY (red), UW228-2 (green), D458 (purple) or D283 (light blue) cells, untreated or stimulated with OA, Chol or a mixture.

In untreated controls, PC1 and PC2 explained 67.3 and 15.7% of the total variance, respectively (83% combined). DAOY and D283 cells occupied distinct regions of the PCA plot, while UW228-2 and D458 showed more intermediate clustering. Pairwise PERMANOVA confirmed the most significant dissimilarity between DAOY and D283 (*F* = 8.18, R^2^ = 0.67) (Figure 2A).

However, under Chol treatment, clustering remained subgroup–specific (PC1 = 35.2%, PC2 = 16.6%; 51.8% total). DAOY and UW228-2 shifted together toward positive PC1 values, indicating a shared Chol-responsive lipid profile, whereas D283 and D458 remained distinct. PERMANOVA supported significant difference between DAOY and D283 subgroups (*F* = 20.97, R^2^ = 0.84) and between D458 and D283 subgroups (*F* = 13.84, R^2^ = 0.78) (Figure 2A).

Following OA treatment, PC1 and PC2 accounted for 56.8 and 19.2% of the variance (76% total). OA induced a significant shift in D458 toward positive PC1 values, distinguishing it from the more similar DAOY and UW228-2 clusters (PERMANOVA: D-458 vs. UW228-2, *F* = 9.58, R^2^ = 0.71) (Figure 2A).

Mixed treatment produced overlapping clusters for DAOY UW228-2 and D458, while D283 remained distinct along negative PC1 (PC1 = 51.5%, PC2 = 17.2%; 68.7% total). PERMANOVA confirmed strong divergence between DAOY and D283 (*F* = 20.85, R^2^ = 0.84) (Figure 2A).

D283 remained the most isolated cell line in the PCA, but its closest neighbour was consistently D458, reflecting their shared G3/G4 phenotype. D283 showed an overall more traditionally aggressive profile, but D458 was particularly sensitive to OA (Figure 2A). Clustering of specific MB groups was further investigated using heat map analysis in Figure 2B, again showing that generally SHH-MBs grouped together, as did G3/G4, with the strongest divergence following Chol stimulation.

### Less aggressive MBs can retain lipid storage in mixed treatment conditions

**Figure 3:**
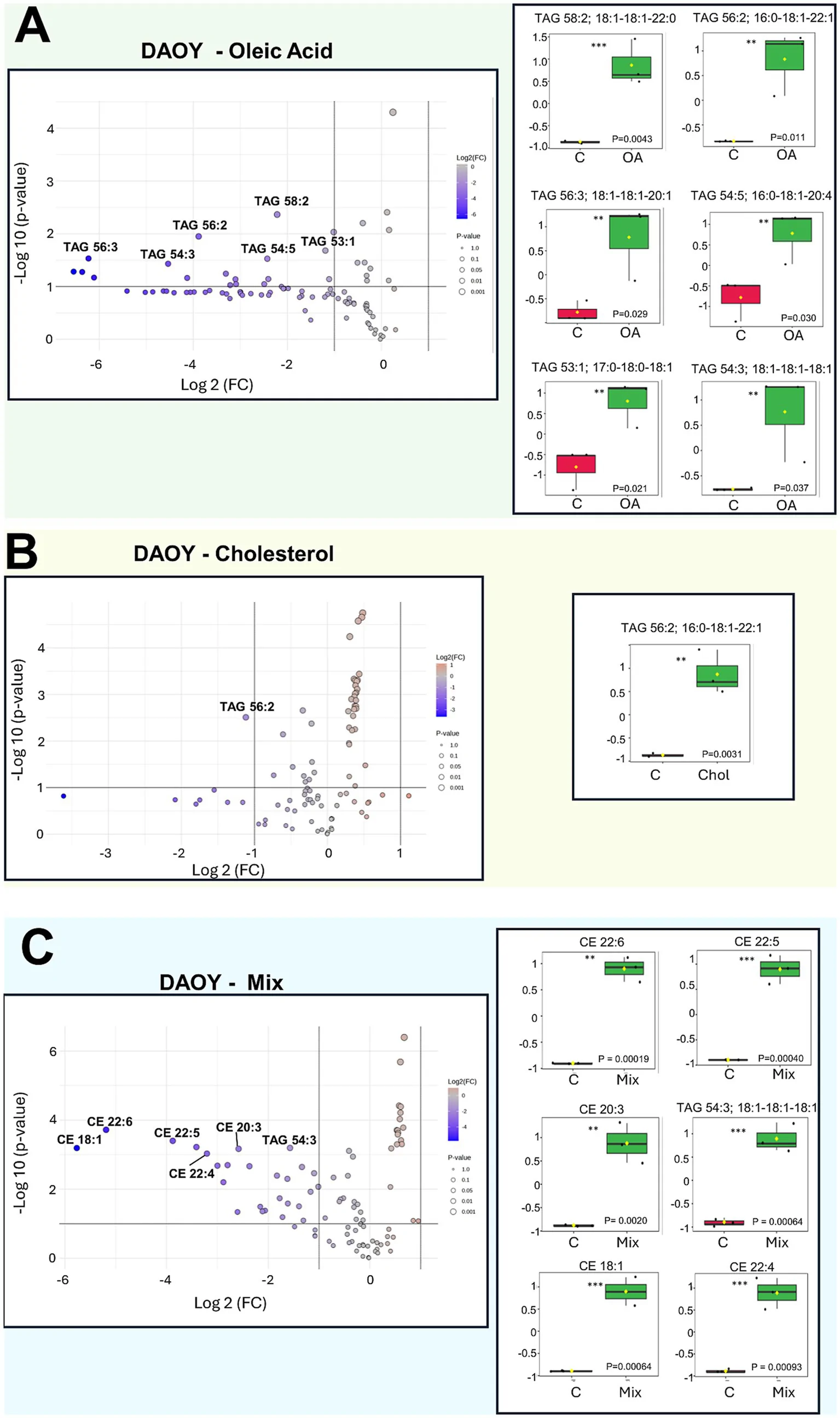
Lipid storage is most enhanced in DAOY cells under mixed conditions. **(A)** Significantly upregulated neutral lipid species detected in lipid droplets isolated from DAOY cells following treatment with OA, as compared to control. **p < 0.05, ***p < 0.01. **(B)** Significantly upregulated neutral lipid species detected in lipid droplets isolated from DAOY cells following treatment with Chol, as compared to control. **p < 0.05. **(C)** Significantly upregulated neutral lipid species detected in lipid droplets isolated from DAOY cells following mixed treatment, as compared to control. **p < 0.05, ***p < 0.01.

**Figure 4:**
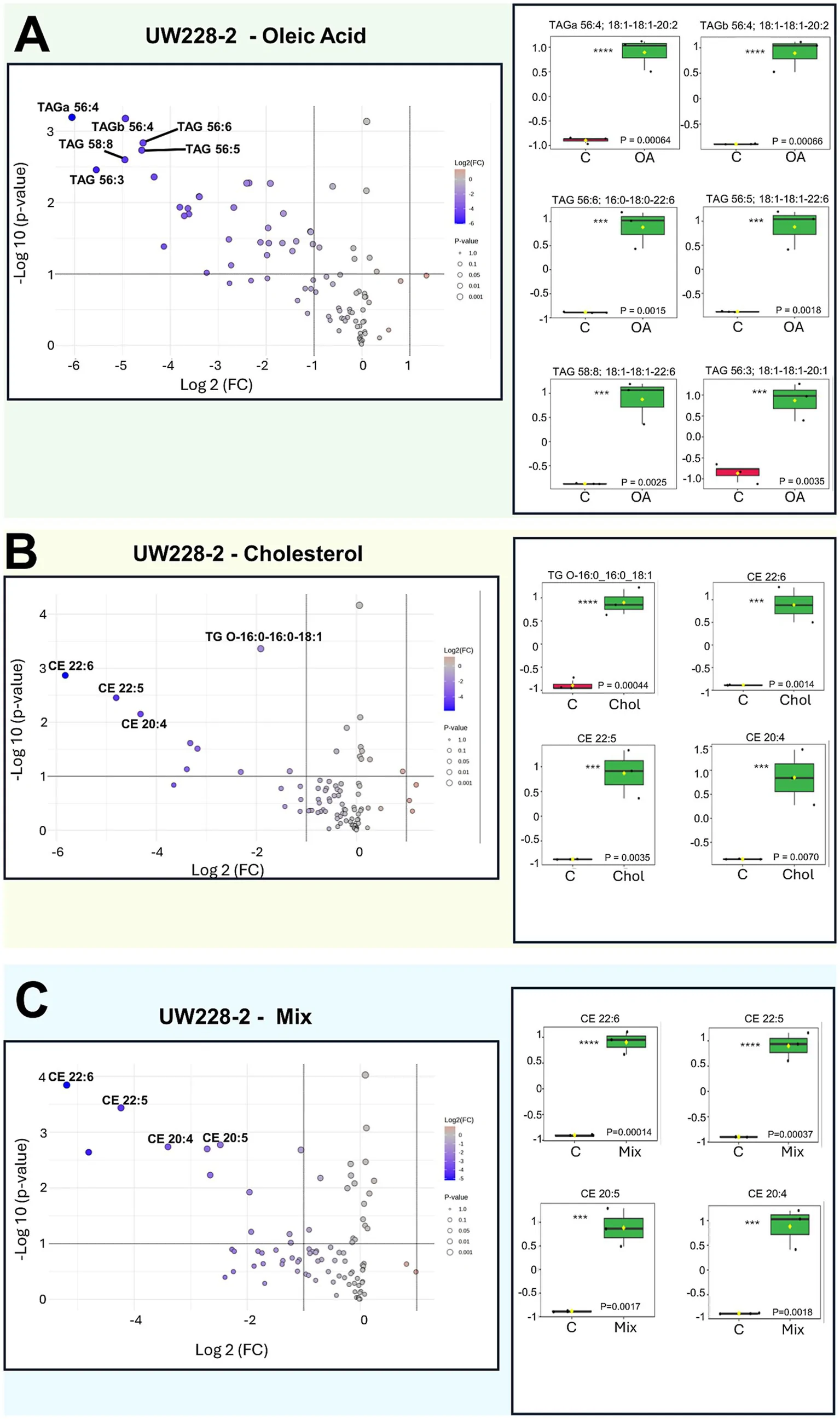
UW228-2 demonstrates the strongest OA-driven TAG upregulation across all MB cell lines. **(A)** Significantly upregulated neutral lipid species detected in lipid droplets isolated from UW228-2 cells following treatment with OA, as compared to control. ***p < 0.01, ****p < 0.001. **(B)** Significantly upregulated neutral lipid species detected in lipid droplets isolated from UW228-2 cells following treatment with Chol, as compared to control. ***p < 0.01, ****p < 0.001. **(C)** Significantly upregulated neutral lipid species detected in lipid droplets isolated from UW228-2 cells following mixed treatment, as compared to control. ***p < 0.01, ****p < 0.001.

**Figure 5:**
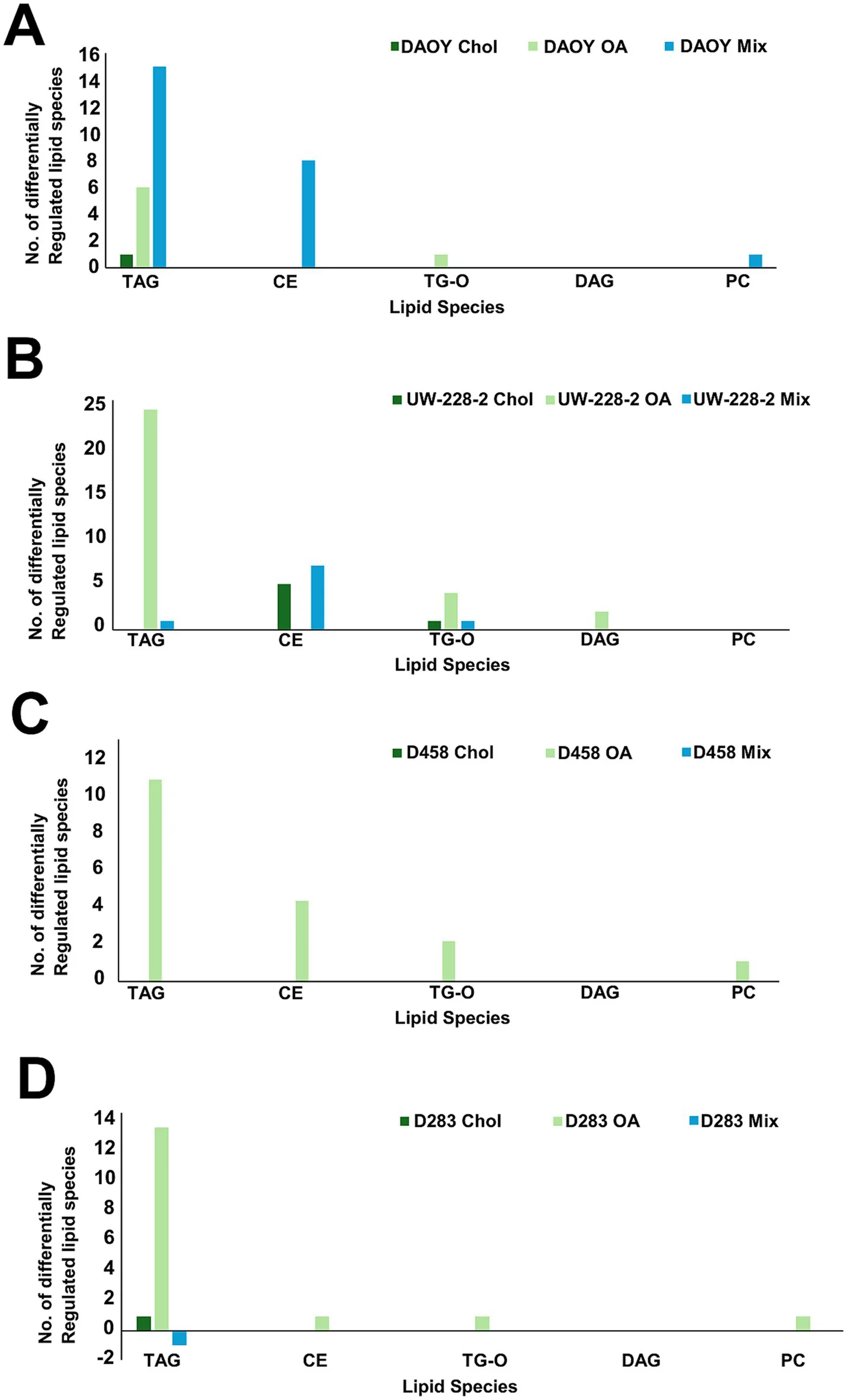
All MB cell lines show significant differences in TAG storage following stimulation conditions, but differences in CEs is limited to SHH MBs. **(A)** Summary of significantly different lipid species in lipid droplets isolated from DAOY cells following treatment with OA, Chol or a mixture as compared to control. The strongest response was observed following mixed treatment. **(B)** Graphical summary of significantly different lipid species in lipid droplets isolated from UW228-2 cells following treatment with OA, Chol or a mixture as compared to control. The strongest response was observed following OA treatment. **(C)** Graphical summary of significantly different lipid species in lipid droplets isolated from D458 cells following treatment with OA, Chol or a mixture as compared to control. The strongest, and only significant, response was observed following OA treatment. **(D)** Graphical summary of significantly different lipid species in lipid droplets isolated from D283 cells following treatment with OA, Chol or a mixture as compared to control. The strongest response was observed following OA treatment.

Whilst PCA and heat map analyses identify global differences in lipid metabolism; it does not reveal which individual species drive this separation. The most important lipid components of LDs are TAGs, CEs, and TGs. To determine which lipid species, contribute to each treatment-dependent shift, volcano plot analysis was performed. OA treatment revealed SHH lines displayed significant induction of long-chain polyunsaturated TAGs, including multiple C56/58 species (Figures 3A, 4A). DAOY displayed a narrow TAG profile, upregulating sixteen species while still showing substantial enrichment of polyunsaturated TAGs (Figure 3A). UW228-2 showed a broader response, upregulating twenty-seven TAG species with strong induction of polyunsaturated Fatty Acid (PUFA)-enriched TAGs (Figure 4A). Both DAOY and UW228-2 significantly upregulated TG/TG-O species relative to their untreated controls (Figures 5A,B).

Interestingly, Chol treatment in DAOY and UW228-2 showed minimal TAG induction, with DAOY upregulating one TAG species, whereas UW228-2 upregulated none (Figures 3B, 4B). Chol overload induced no other significantly upregulated species in DAOY (Figure 5A), whereas UW228-2, which showed no CE induction under OA, upregulated five CE species under Chol treatment (Figure 5B).

Mixed treatment further highlights SHH line differences; DAOY was able to upregulate eight CE species under mixed conditions, despite the lack of CE in Chol or OA conditions (Figures 3C, 5A), whereas UW228-2 strengthened its Chol-driven CE profile, upregulating seven CE species (Figures 4C, 5B). DAOY was able to maintain its OA-driven TAG profile, upregulating sixteen TAG species, whereas UW228-2 only upregulated four TAG species (Figures 5A, 5B). UW228-2 was able to upregulate TG/TG-O species across all treatment groups (Figure 5B). Taken together, these data indicate that less aggressive MB cell lines retain a substantial capacity for lipid storage under mixed treatment conditions, maintaining TAG and/or CE accumulation despite concurrent lipid challenges.

### G3/G4-MB cells display an OA-dependent storage profile

**Figure 6:**
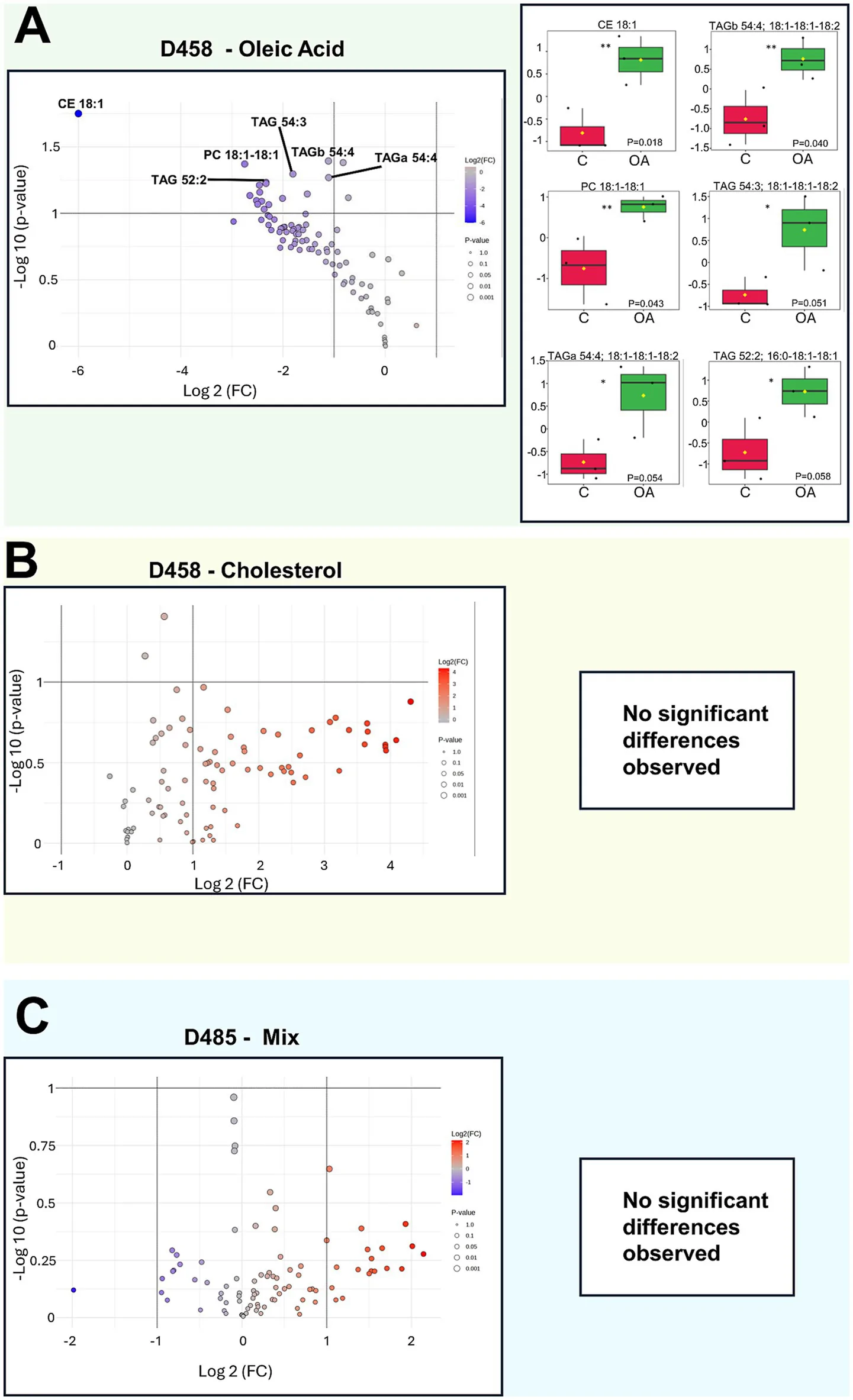
D458 cells show a uniquely selective OA-driven lipid storage profile. **(A)** Significantly upregulated neutral lipid species detected in lipid droplets isolated from D458 cells following treatment with OA, as compared to control. *p < 0.1, **p < 0.05. **(B)** Volcano plot showing detected neutral lipid species in isolated lipid droplets from D458 cells following stimulation with Chol, as compared to control. No species were found to be above significance thresholds. **(C)** Volcano plot showing detected neutral lipid species in isolated lipid droplets from D458 cells following mixed stimulation, as compared to control. No species were found to be above significance thresholds.

**Figure 7:**
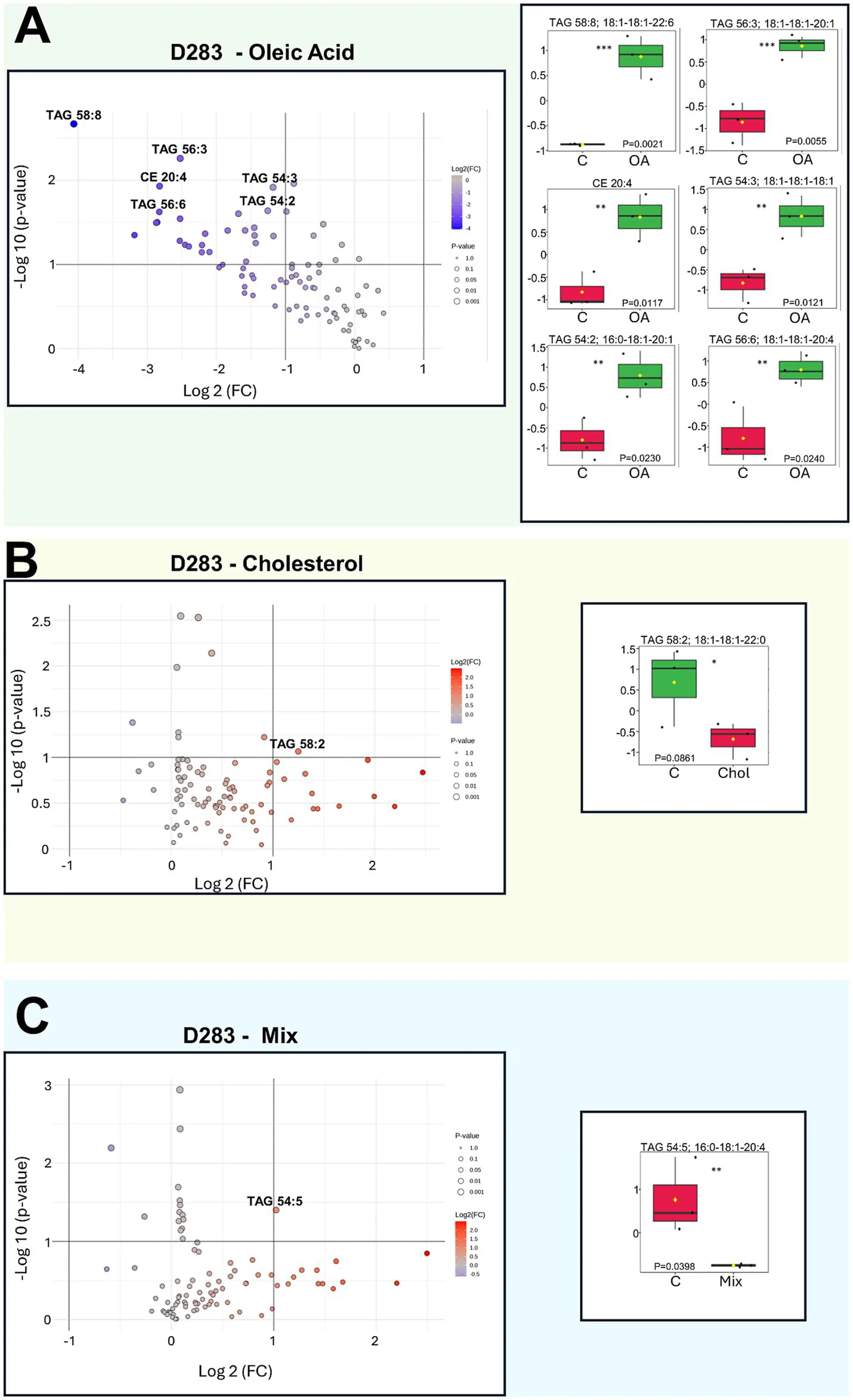
D283 cells present a narrow OA-driven TAG profile. **(A)** Significantly upregulated neutral lipid species detected in lipid droplets isolated from D283 cells following treatment with OA, as compared to control. **p < 0.05, ***p < 0.01. **(B)** Significantly upregulated neutral lipid species detected in lipid droplets isolated from D283 cells following treatment with Chol, as compared to control. *p < 0.1. **(C)** Significantly upregulated neutral lipid species detected in lipid droplets isolated from D283 cells following mixed treatment, as compared to control. **p < 0.05.

Oleic acid is normally present in the cerebrospinal fluid (CSF) and is one of the major monounsaturated fatty acids in the central nervous system. To help understand subgroup-specific differences against the more aggressive subtype, volcano plots were generated for D458, a MYC-driven G3-MB (Figure 6), and D283, a G3/G4-MB cells (Figure 7).

Both D458 and D283 exhibited a narrower, selectively OA-induced TAG storage profile (Figures 6A, 7A), characterised by long-chain TAG induction, with 18:1-rich families. D458 displayed the most restrictive TAG storage profile, upregulating only ten species (Figure 5C) as opposed to eighteen in D283 (Figure 5D). Several TAG backbones were shared between the two cell lines, such as TAG 54:3, 56:3, 58:6, and 58:8, but with generally weaker induction in D283. In contrast to the SHH subgroups, both D283 and D458 could upregulate CE species under OA treatment. A narrow TG/TG-O signal was present in both (Figures 6A, 7A).

Under Chol treatment, D458 showed a collapse in lipid storage, with no significant lipid changes (Figure 6B). D283 exhibited a similar phenotype, except for a single upregulated TAG species (TAG 58:2; 18:1–18:1–22:0) that was not induced under OA alone (Figure 7B).

Mixed treatment provided a key subgroup-specific pattern. The OA-induced lipid profile was entirely lost, with no significantly upregulated TAG, CE or TG-O species. D283 downregulated one TAG species (TAG 54:5; 16:0–18:1–20:4) (Figure 7C), which was upregulated in OA (Figure 7A).

Using all volcano plots, DAOY and UW228-2 lines shared seven overlapping lipid species, but UW228-2 overlapped more extensively with the more aggressive subgroup. UW228-2 shared twelve lipid species with D458 and eighteen with D283, consistent with UW228-2’s intermediate PCA position.

The number of cell line-specific species was highest in DAOY, which upregulated twenty-one unique species, comprising eighteen TAGs, one TG-O, CE, and PC. UW228-2 displayed twelve unique species, including eight TAGs and two TG-O/ DAGs. In contrast, the more aggressive G3/G4 lines displayed far fewer unique storage lipids: D458 contained only three unique species, one TAG, CE and TG-O. In comparison, D283 upregulated four distinct species, comprising one TAG, two CEs, and one TG-O. This distribution indicated that G3/G4 cells exhibit a more restricted, selective lipid profile (Figure 5).

**Figure 8:**
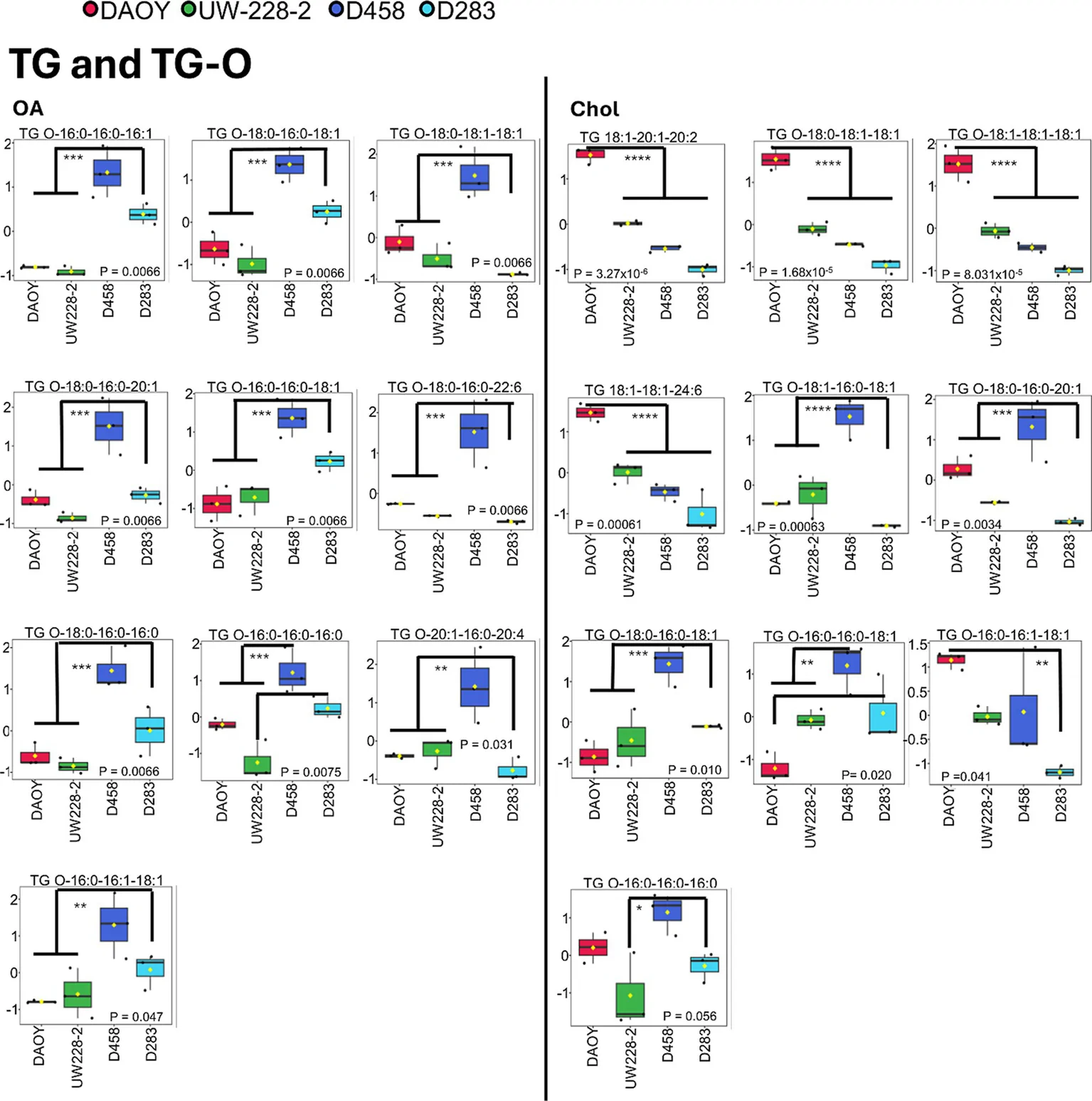
A distinct TG enrichment pattern is seen in DAOY and D458 cells following stimulation with OA and Chol. Significantly altered TG and oxidised TG species detected in DAOY and D458 cells as compared across all four MB cell lines following treatment with OA or Chol. Significant differences were assessed using one-way ANOVA (FDR *p* < 0.1) with Tukey’s post-hoc comparisons test. *p < 0.1, **p < 0.05, ***p < 0.01, ****p < 0.001.

**Figure 9:**
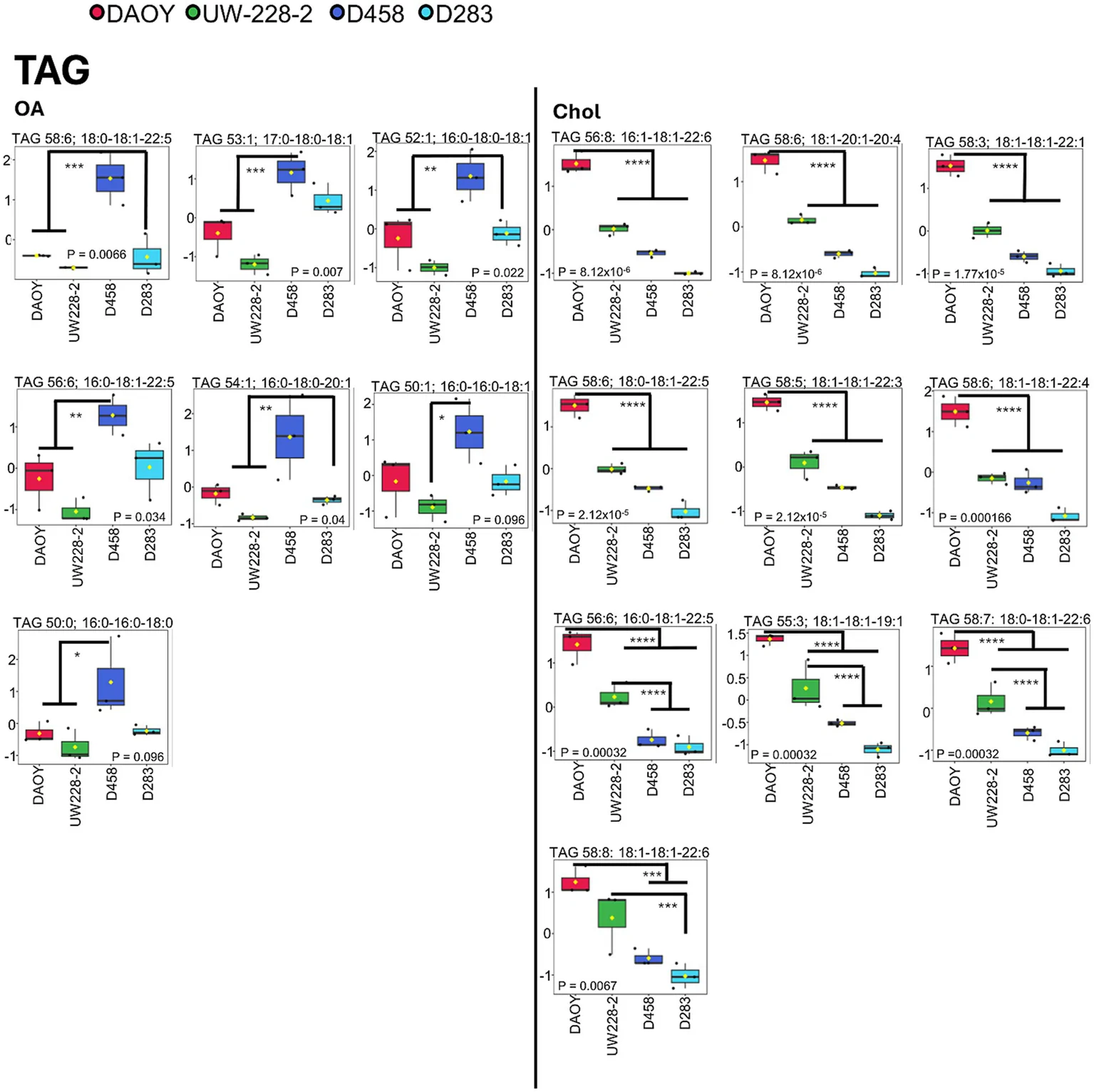
Upregulation of TAG species following Chol treatment is isolated to SHH MBs. Significantly altered TAG species detected in all four MB cells following treatment with OA or Chol. Significant differences were assessed using One-Way ANOVA (FDR *p* < 0.1) with Tukey’s post-hoc comparisons test. *p < 0.1, **p < 0.05, ***p < 0.01, ****p < 0.001.

**Figure 10:**
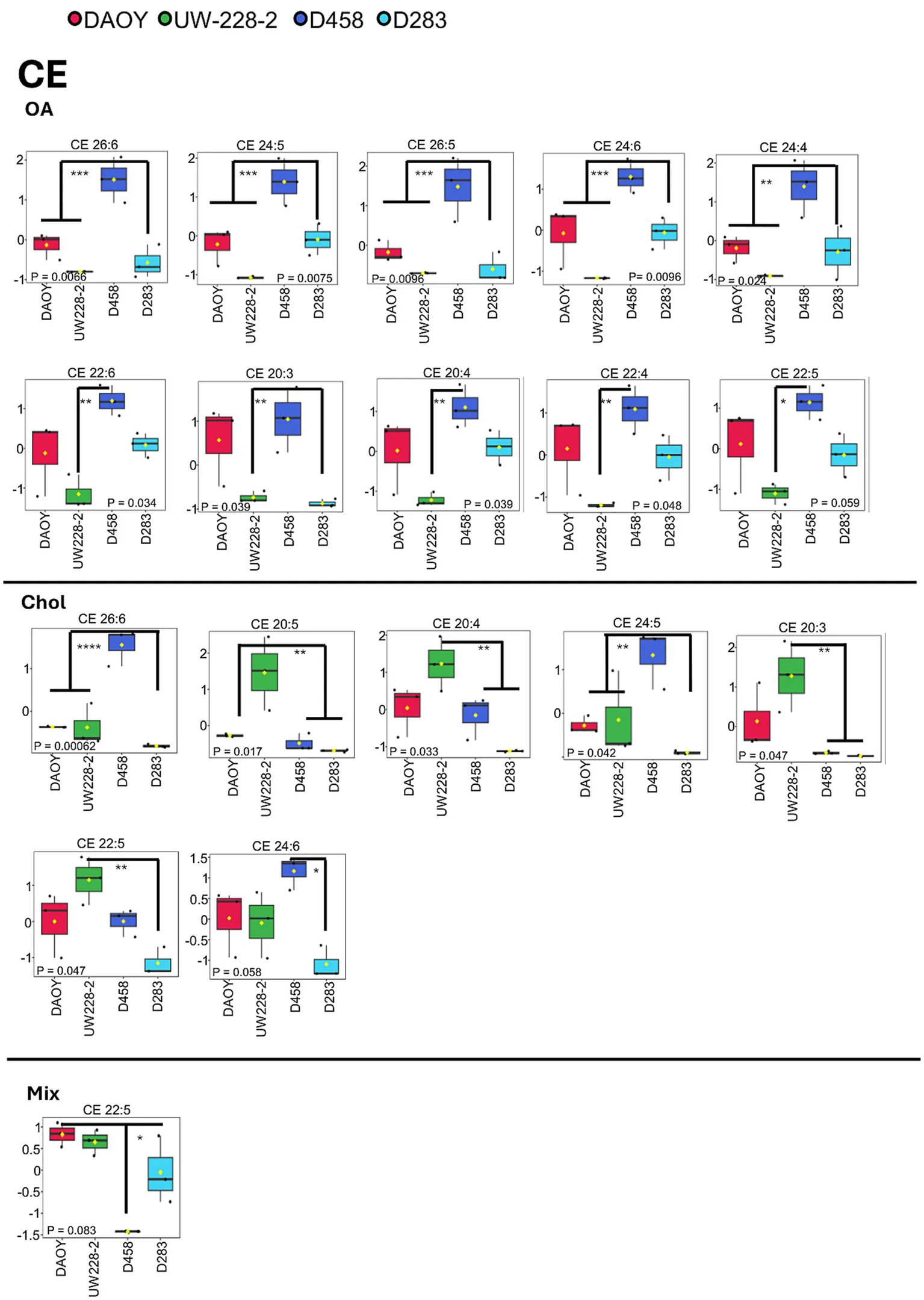
Mixed treatment abolishes the CE storage hierarchy in MB cells. Significantly altered CE species detected in all four MB cells following treatment with OA, Chol or mixed. Significant differences were assessed using One-Way ANOVA (FDR *p* < 0.1) with Tukey’s post-hoc comparisons test. *p < 0.1, **p < 0.05, ***p < 0.01, ****p < 0.001.

Volcano plots were used to describe within-cell-line responses in comparison to each cell line’s untreated control. To compare lipid species across all four MB cell lines and treatments, one-way ANOVA with Tukey’s correction was performed (Figures 8–10). Taken together, these data indicate that G3/G4-MB cells exhibit a highly selective and OA-dependent lipid storage program that is largely abolished by Chol or mixed lipid exposure.

### D458-MB cells upregulate TG-O species following both Chol and OA treatment

Chol is highly abundant in the brain and a major constituent of LDs. Chol treatment revealed a distinct TG species pattern that separated into a SHH and a D458 cluster (Figure 8).

In the SHH cluster, DAOY selectively upregulated long-chain unsaturated TG/TGO species (18:1-, 20:1-, 20:2-, and 24:6-containing backbones), representing a highly unsaturated storage profile. The SHH cluster followed the hierarchy of DAOY>UW228-2 > D458 > D283, showing a subgroup-specific ability to store unsaturated TG species under Chol treatment (Figure 8). D458 dominated a separate cluster enriched in shorter saturated/mixed chain TG/TG-O species, particularly 16C-rich backbones, with no clear hierarchy among the other three cell lines.

Under OA treatment, TG-O species were significantly increased in all cell lines, displaying a clear G3/G4 dominant hierarchy: D458 > D283 > DAOY>UW228-2 (Figure 8). D458 displayed the largest cell line storage profile of TG/TG-O storage, D283 showed a similar, more modest profile on select TG species, and both SHH lines display a minimal TG storage program (Figure 8).

Across all four MB cell lines, no TG or TG-O species reached statistical significance under mixed treatment.

### Cholesterol induces a SHH-dominant TAG profile

Chol treatment produced a highly consistent and subgroup-specific TAG response (Figure 9). A total of ten TAG species were differentially upregulated in response to Chol, including long-chain PUFA TAG species (TAG 58 and 56). All Chol-related upregulation of TAG species follows the same expression pattern of DAOY>UW228-2 > D458 > D283, showing that SHH lines are most responsive to cholesterol loading, specifically evident in DAOY. D283 consistently exhibited the lowest changes regarding Chol-induced TAG storage (Figure 9).

Under OA treatment, TAG regulation showed a broader but less pronounced expression profile than under Chol. Significant species across cell lines included shorter chain PUFA TAGs (TAG 50–56), in contrast to the predominantly longer TAG species seen upregulated in DAOY under Chol treatment. OA TAG upregulation followed a reverse order: D458 > D283 > DAOY>UW228-2 (Figure 9).

No TAG species were significantly differentially upregulated across mixed treatments.

### Mixed treatment causes cholesterol esters storage collapse

TAG and TG profiles largely reflect FA-driven storage. Under OA treatment, D458 consistently exhibited the strongest CE response across all cell lines, significantly upregulating both longer chain CEs (CE 24/26), as well as shorter chain CE species (CE 20/22). DAOY and D283 appear to primarily upregulate shorter chain CEs, while UW228-2 consistently showed minimal CE storage (Figure 10).

Under Chol treatment, D458 again showcased its ability to store longer chain CE species, and unlike OA-treated D458, shorter chain CE signalling collapsed in comparison to the other MB cell lines. In contrast, UW228-2, which showed no CE induction under OA, displayed a moderate CE induction under Chol, indicating a Chol-dependent sterol storage mechanism. D283 showed minimal upregulation of CE species (Figure 10).

In the mixed treatment, all differences observed in OA and Chol treatment was abolished. Only one CE species was significantly different across all cell lines (CE 22:5). This species was downregulated in D458 despite being upregulated in both D458 OA and Chol (Figure 10).

Taken together, these data indicate that mixed lipid exposure abolishes the distinct CE storage phenotypes observed under OA and Chol treatment, resulting in a marked collapse of CE accumulation across MB subgroups.

## Discussion

This study reveals the effect of OA and Chol overload in MB cells. These conditions were used because they represent the predominant lipid species encountered by MB cells in the brain microenvironment and are characterised as key drivers for LD biogenesis, in the form of TAGs and CEs (Kraemer et al., 2013; Rohwedder et al., 2014).

OA is one of the most abundant monounsaturated fatty acids in the CNS. It is found in various foods, being up taken by neurons, as well as being actively produced and metabolised by astrocytes (Tabernero et al., 2001). The role of OA in cancer is tumour-dependent, with both pro- and anti—tumorigenic effects reported (Deng et al., 2023).

Chol is the most enriched lipid in the brain, accounting for 20–25% of the body’s total cholesterol (Jin et al., 2019), where it is essential for membrane formation, microdomain organisation, and synaptic function. Importantly, Chol is unable to cross the blood–brain barrier, thus brain concentrations are synthesised on site.

A key observation was that although SHH-MB cells upregulate a greater number of TAG species in OA and Mix conditions (Figures 3A, 4A), the G3/G4-MB lines showed a more selective, but intensified, OA-dependent lipid storage program. This narrow program observed suggests the existence of lipid processing pathways in G3/G4-MBs that are not present in SHH cells. Similar studies describe G3/G4-MBs as heavily lipogenic with elevated fatty acid synthase (FASN) and acetyl-CoA carboxylase 1 (ACC1) driving basal FA storage (Tech and Gershon, 2015). This evidence highlights that the unique lipid synthesis pathways previously observed in literature may be partnered with distinctive processing pathways that must be further characterised.

Another key feature of G3/G4 lipid storage was the unique ability to upregulate CEs under OA monotreatment, offering a potentially targetable adaptation, pending further exploration (Figures 5C, 5D) (Comer et al., 2025). Literature has shown non-rewired cells are unable to store excess FAs as CEs, whilst cancerous tissue such as GBM can (Kou et al., 2022). This ability could act as a hallmark of G3/G4-MBs, linking upregulated CE storage with aggressive tumour nature (Yu and Aboud, 2024).

Chol overload also drives LD formation, being esterified into CEs, but also by altering the ER membrane properties to activate LD biogenesis (Ralhan et al., 2021; Kumari et al., 2023). In contrast to OA responses, G3/G4-MB cells showed no significant effects in TAG storage mechanisms under Chol monotreatment (Figures 5C, 5D). Thus, highlighting a unique G3/G4 Chol response that may have potential for further interrogation.

Notably, only UW228-2 responded as expected to Chol treatment, through upregulation of CEs. However, whilst D458 and D283 cells did not appear to respond to the treatment, they did maintain an already increased basal expression of CEs, likely generated from the increased Chol biosynthesis previously detected in these cells (Comer et al., 2025).

Expression of TG-O species, or lack thereof, could provide an insight into the oxidative phenotype of the cells. Only UW228-2 upregulated TG-O species under all treatments (Figure 5B). However, ANOVA analysis detected high TG-O concentrations in D458 cells but not in D283 cells (Figures 5C,D, 8). This may suggest that G3/G4 cells are oxidating more fatty acids, supported by literature demonstrating active anti-oxidant mechanisms in these cells (Marabitti et al., 2022). Further validation of this may identify a targetable characteristic of aggressive MBs moving forward.

Following mixed treatment, SHH cells retained the ability to package lipids observed through the upregulation of TAGs and CEs, highlighting the resilient and adaptable lipid storage mechanism. Conversely, D458 and D283 cells demonstrated a collapse in lipid storage, as Chol appeared to antagonise the robust OA response (Figure 5). This further highlights the divergent lipid response pathways between different MB subgroups and thus the potential for further exploration in the search for novel therapeutics.

Literature has previously shown that aggressive MBs overexpress SOAT1 (Comer et al., 2025) and higher expression of this enzyme has previously been linked to invasive behaviour in G3/G4 tumours (Albert et al., 2021). Additionally, as previously stated Chol has limited capacity for blood–brain barrier permeability and G3/G4 MBs have adapted to account for this, synthesising their own Chol at an increased rate. As such, a reasonable explanation for minimal response to exogenous Chol could relate to a higher activity of the intrinsic synthesis pathway observed frequently in literature.

Whilst the data presented here discusses promising avenues for further research, it is important to highlight the limitations of the presented results. This study provides a wide view of the lipidomic profile in MB cells to support further characterisation of the disease. As such, we utilised more lenient statistical thresholds than typical to encapsulate this expanded viewpoint, whilst still remaining within relative confidence and supporting any observations with trends discovered by other researchers (Comer et al., 2025; Yu and Aboud, 2024; Bernardi et al., 2026; Cheng et al., 2020). Moreover, the data presented is drawn from lipid droplets isolated from commercially available cell lines of MB. Future validation should therefore seek to explore the observed effects, and any further effects, in more advanced 3D, patient-derived and *in vivo* models.

In summary, these data reveal subgroup differences in lipid metabolism within MBs. Most promisingly, G3/G4-MBs unique response to Chol and Mix treatment highlights a potential specific dependence on internal Chol biosynthesis. Providing rationale for further exploration to elucidate the critical role internal Chol biosynthesis is playing in the high proliferation and metastasis of G3/G4-MBs. Ultimately, this study serves as a broad summary on the composition of neutral lipid droplets in MBs, highlighting promising avenues for further exploration within this field.

## Data Availability

The raw data supporting the conclusions of this article will be made available by the authors, without undue reservation.

## Footnotes

1
https://www.metaboanalyst.ca

## Supplementary material

SUPPLEMENTARY FIGURE S1 Representative fluorescent images of DAOY cells untreated or treated with OA, Chol or mix stained with DAPI (Blue) and Nile Red (Red). Taken at 20x magnification, scale bar = 100 μm.

SUPPLEMENTARY FIGURE S2 Representative fluorescent images of UW228-2 cells untreated or treated with OA, Chol or mix stained with DAPI (Blue) and Nile Red (Red). Taken at 20x magnification, scale bar = 100 μm.

SUPPLEMENTARY FIGURE S3 Representative fluorescent images of D458 cells untreated or treated with OA, Chol or mix stained with DAPI (Blue) and Nile Red (Red). Taken at 20x magnification, scale bar = 100 μm.

SUPPLEMENTARY FIGURE S4 Representative fluorescent images of D283 cells untreated or treated with OA, Chol or mix stained with DAPI (Blue) and Nile Red (Red). Taken at 20x magnification, scale bar = 100 μm.

## References

[ref1] Albert T. K. Interlandi M. Sill M. Graf M. Moreno N. Menck K. . (2021). An extracellular vesicle-related gene expression signature identifies high-risk patients in medulloblastoma. Neuro-Oncology 23, 586–598. doi: 10.1093/neuonc/noaa254, 33175161 PMC8041350

[ref2] Bernardi F. Torrejon J. Basili I. van Ommeren R. Marsaud V. Yu H. . (2026). Multiomic integration reveals tumoral heterogeneity of lipid dependence within lethal group 3 medulloblastoma. Cancer Cell 44, 383–404.e18. doi: 10.1016/j.ccell.2025.12.012, 41544627 PMC13418108

[ref3] Bingi T. Cotton K. Comer C. Niklison-Chirou M. V. (2024). Are lipid droplets the picnic basket of brain tumours? Cell Death Discovery 10:31. doi: 10.1038/s41420-024-01797-8, 38228582 PMC10791621

[ref4] Cheng X. Geng F. Pan M. Wu X. Zhong Y. Wang C. . (2020). Targeting DGAT1 ameliorates glioblastoma by increasing fat catabolism and oxidative stress. Cell Metab. 32, 229–242.e8. doi: 10.1016/j.cmet.2020.06.002, 32559414 PMC7415721

[ref5] Comer C. Cotton K. Edwards C. Dai X. Badodi S. Buccafusca R. . (2025). Simvastatin suppresses spinal cord metastasis of medulloblastoma at clinically significant doses. Cell Death Dis. 16:527. doi: 10.1038/s41419-025-07829-0, 40664675 PMC12263873

[ref6] Deng B. Kong W. Suo H. Shen X. Newton M. A. Burkett W. C. . (2023). Oleic acid exhibits anti-proliferative and anti-invasive activities via the PTEN/AKT/mTOR pathway in endometrial Cancer. Cancer 15:5407. doi: 10.3390/cancers15225407, 38001668 PMC10670880

[ref7] Fernández-García P. Malet-Engra G. Torres M. Hanson D. Rosselló C. A. Román R. . (2023). Evolving diagnostic and treatment strategies for pediatric CNS tumors: the impact of lipid metabolism. Biomedicine 11:1365. doi: 10.3390/biomedicines11051365, 37239036 PMC10216525

[ref8] Huang R. Lu X. Sun X. Wu H. (2024). Metabolomic profiling of childhood medulloblastoma: contributions and relevance to diagnosis and molecular subtyping. J. Cancer Res. Clin. Oncol. 150:471. doi: 10.1007/s00432-024-05990-1, 39441459 PMC11499513

[ref9] Jin U. Park S. J. Park S. M. (2019). Cholesterol metabolism in the brain and its association with Parkinson's disease. Exp Neurobiol 28, 554–567. doi: 10.5607/en.2019.28.5.554, 31698548 PMC6844833

[ref10] Kou Y. Geng F. Guo D. (2022). Lipid metabolism in glioblastoma: from De novo synthesis to storage. Biomedicine 10:1943. doi: 10.3390/biomedicines10081943, 36009491 PMC9405736

[ref11] Kraemer F. B. Khor V. K. Shen W. J. Azhar S. (2013). Cholesterol ester droplets and steroidogenesis. Mol. Cell. Endocrinol. 371, 15–19. doi: 10.1016/j.mce.2012.10.012, 23089211 PMC3584206

[ref12] Kumari R. M. Khatri A. Chaudhary R. Choudhary V. (2023). Concept of lipid droplet biogenesis. Eur. J. Cell Biol. 102:151362. doi: 10.1016/j.ejcb.2023.151362, 37742390 PMC7615795

[ref13] Lee B. Mahmud I. Pokhrel R. Murad R. Yuan M. Stapleton S. . (2022). Medulloblastoma cerebrospinal fluid reveals metabolites and lipids indicative of hypoxia and cancer-specific RNAs. Acta Neuropathol. Commun. 10:25. doi: 10.1186/s40478-022-01326-7, 35209946 PMC8867780

[ref14] Marabitti V. Giansanti M. De Mitri F. Gatto F. Mastronuzzi A. Nazio F. (2022). Pathological implications of metabolic reprogramming and its therapeutic potential in medulloblastoma. Front. Cell Dev. Biol. 10, 1–19. doi: 10.3389/fcell.2022.1007641, 36340043 PMC9627342

[ref15] Martin-Perez M. Urdiroz-Urricelqui U. Bigas C. Benitah S. A. (2022). The role of lipids in cancer progression and metastasis. Cell Metab. 34, 1675–1699. doi: 10.1016/j.cmet.2022.09.023, 36261043

[ref16] Medina J. Borreggine R. Teav T. Gao L. Ji S. Carrard J. . (2023). Omic-scale high-throughput quantitative LC-MS/MS approach for circulatory lipid phenotyping in clinical research. Anal. Chem. 95, 3168–3179. doi: 10.1021/acs.analchem.2c02598, 36716250

[ref17] Northcott P. A. Hielscher T. Dubuc A. Mack S. Shih D. Remke M. . (2011). Pediatric and adult sonic hedgehog medulloblastomas are clinically and molecularly distinct. Acta Neuropathol. 122, 231–240. doi: 10.1007/s00401-011-0846-7, 21681522 PMC4538327

[ref18] Ralhan I. Chang C. L. Lippincott-Schwartz J. Ioannou M. S. (2021). Lipid droplets in the nervous system. J. Cell Biol. 220:7. doi: 10.1083/jcb.202102136, 34152362 PMC8222944

[ref19] Rohwedder A. Zhang Q. Rudge S. A. Wakelam M. J. (2014). Lipid droplet formation in response to oleic acid in Huh-7 cells is mediated by the fatty acid receptor FFAR4. J. Cell Sci. 127, 3104–3115. doi: 10.1242/jcs.145854, 24876224

[ref20] Shaw C. S. Clark J. A. Shepherd S. O. (2013). HSL and ATGL: the movers and shakers of muscle lipolysis. J. Physiol. 591, 6137–6138. doi: 10.1113/jphysiol.2013.265199, 24339152 PMC3892467

[ref21] Shi Z. Yang C. Xu X. Wu W. Bao L. (2025). Plasma Lipidomics Profiling to Identify Signatures of Pediatric Medulloblastoma. Beijing, China: Elsevier BV. doi: 10.1007/s12094-025-04008-7 40833672

[ref22] Tabernero A. Lavado E. M. Granda B. Velasco A. Medina J. M. (2001). Neuronal differentiation is triggered by oleic acid synthesized and released by astrocytes. J. Neurochem. 79, 606–616. doi: 10.1046/j.1471-4159.2001.00598.x, 11701764

[ref23] Talari N. K. Mattam U. Meher N. K. Paripati A. K. Mahadev K. Krishnamoorthy T. . (2023). Lipid-droplet associated mitochondria promote fatty-acid oxidation through a distinct bioenergetic pattern in male Wistar rats. Nat. Commun. 14:766. doi: 10.1038/s41467-023-36432-0, 36765117 PMC9918515

[ref24] Tech K. Gershon T. R. (2015). Energy metabolism in neurodevelopment and medulloblastoma. Transl. Pediatr. 4, 12–19. doi: 10.3978/j.issn.2224-4336.2015.01.03 26835355 PMC4729065

[ref25] Wang G. Sun B. Liu H. Hu M. Xu H. Li H. . (2025). Interactions between lipid droplets and mitochondria in metabolic diseases. Lipids Health Dis. 24:357. doi: 10.1186/s12944-025-02759-4, 41219930 PMC12607196

[ref26] Yu N. Aboud O. (2024). The Lipidomic signature of glioblastoma: a promising frontier in Cancer research. Cancer 16:1089. doi: 10.3390/cancers16061089, 38539424 PMC10968728

[ref27] Zadoorian A. Du X. Yang H. (2023). Lipid droplet biogenesis and functions in health and disease. Nat. Rev. Endocrinol. 19, 443–459. doi: 10.1038/s41574-023-00845-0, 37221402 PMC10204695

