# Supplementary material for: Lipidomic analysis of isolated lipid droplets reveals potential metabolic pathway differences between medulloblastoma subgroups
Source: Front Neurosci. 2026 Sep 1;20:1863712. doi: 10.3389/fnins.2026.1863712 (PMC13574944; doi:10.3389/fnins.2026.1863712)

Supp Table 1: Pairwise PERMANOVA results of lipidomic variance between medulloblastoma cell lines under different treatment conditions taken from PCA analysis.

Treatment	Comparison	F-Value	R^2	P-Value
**Control**	D283 vs D458	0.883	0.181	0.60
	D283 vs DAOY	8.18	0.67	0.10
	D283 vs UW228-2	2.12	0.35	0.30
	D458 vs DAOY	1.29	0.24	0.40
	D458 vs UW228-2	1.53	0.28	0.40
	DAOY vs UW228-2	160.23	0.98	0.10
**Cholester ol**	D283 vs D458	13.82	0.78	0.12
	D283 vs DAOY	20.97	0.84	0.12
	D283 vs UW228-2	6.66	0.63	0.12
	D458 vs DAOY	13.84	0.78	0.12
	D458 vs UW228-2	3.22	0.45	0.12
	DAOY vs UW228-2	0.731	0.16	0.60
**Oleic Acid**	D283 vs D458	6.96	0.64	0.10
	D283 vs DAOY	1.27	0.24	0.40
	D283 vs UW228-2	3.05	0.43	0.20
	D458 vs DAOY	2.22	0.36	0.30
	D458 vs UW228-2	9.58	0.71	0.10
	DAOY vs UW228-2	0.62	0.13	0.60
**Mix**	D283 vs D458	1.60	0.29	0.20
	D283 vs DAOY	20.85	0.84	0.10
	D283 vs UW228-2	4.29	0.52	0.10
	D458 vs DAOY	1.33	0.30	0.30
	D458 vs UW228-2	0.9	0.18	0.50
	DAOY vs UW228-2	0.26	0.06	0.70

Cotton K Supp Figure 1

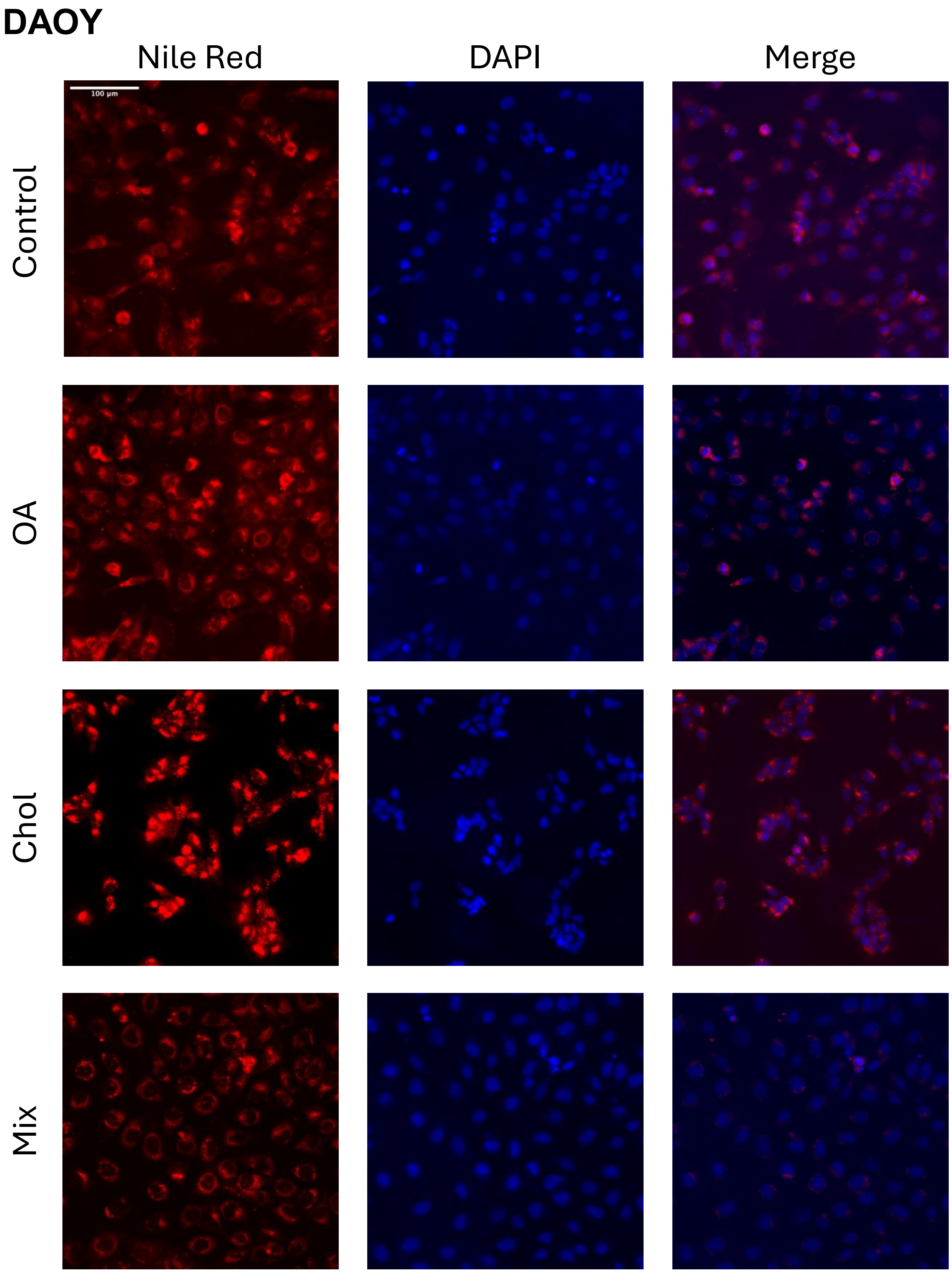

Cotton K Supp Figure 2

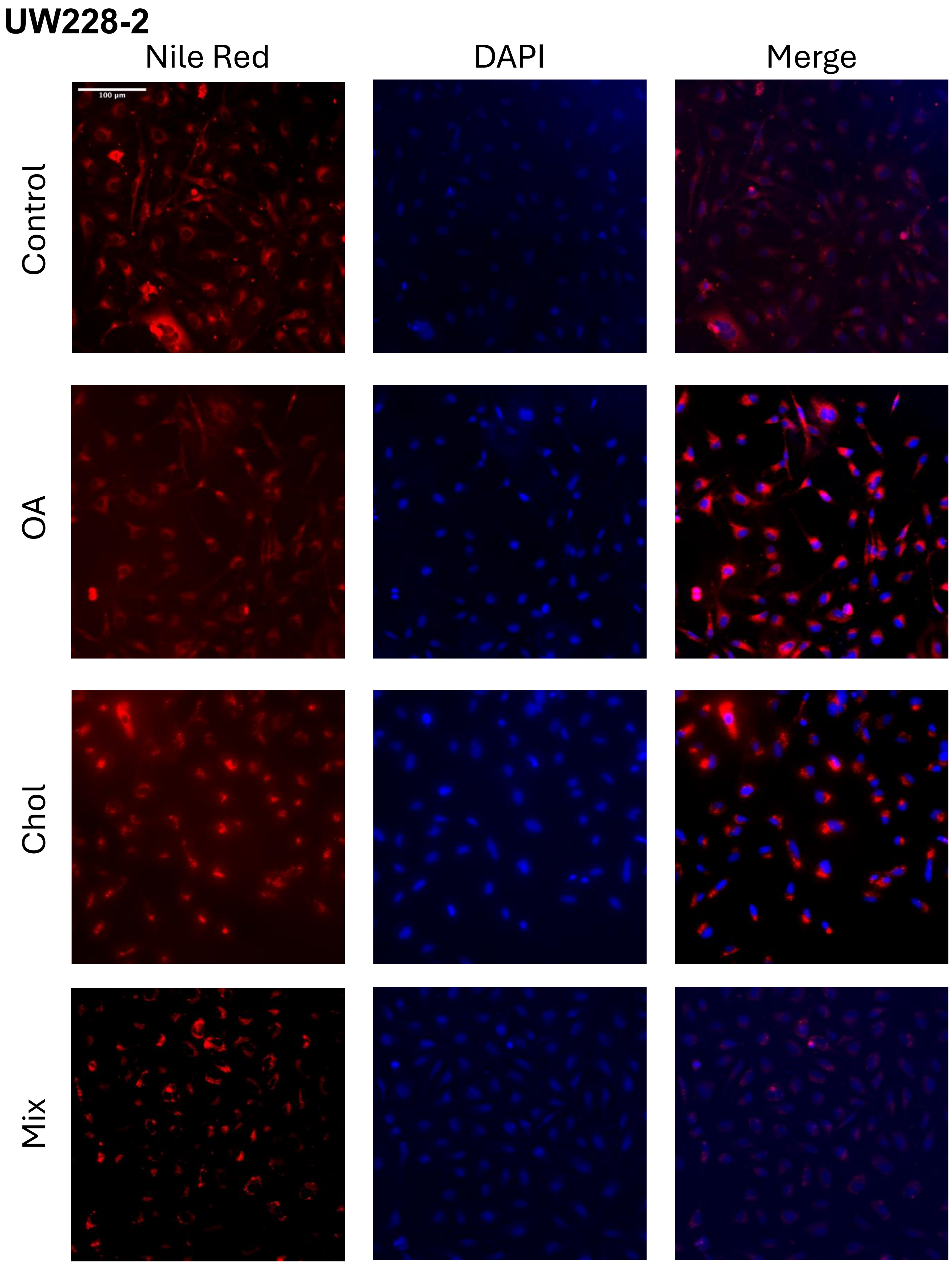

Cotton K Supp Figure 3

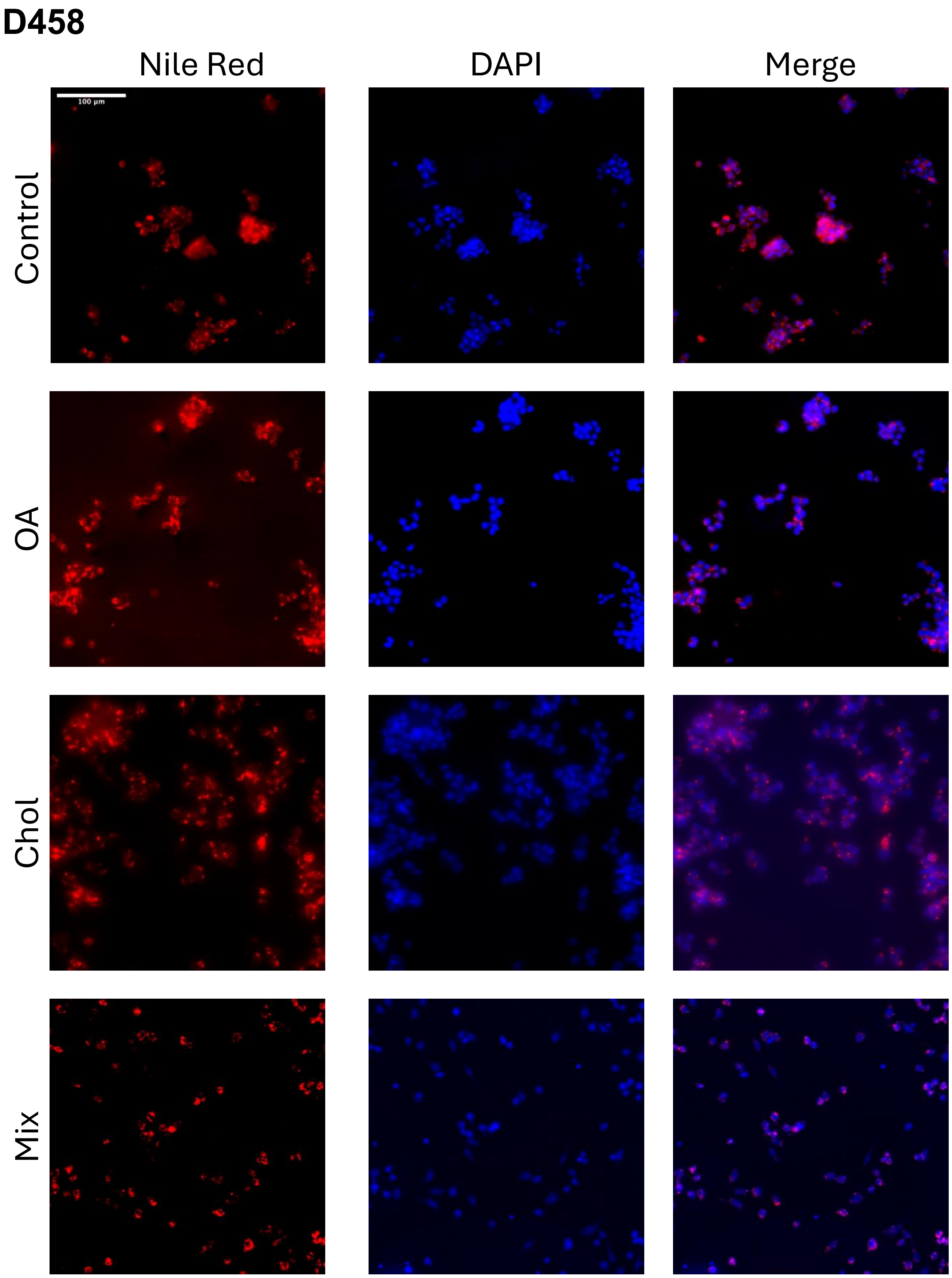

Cotton K Supp Figure 4

D283

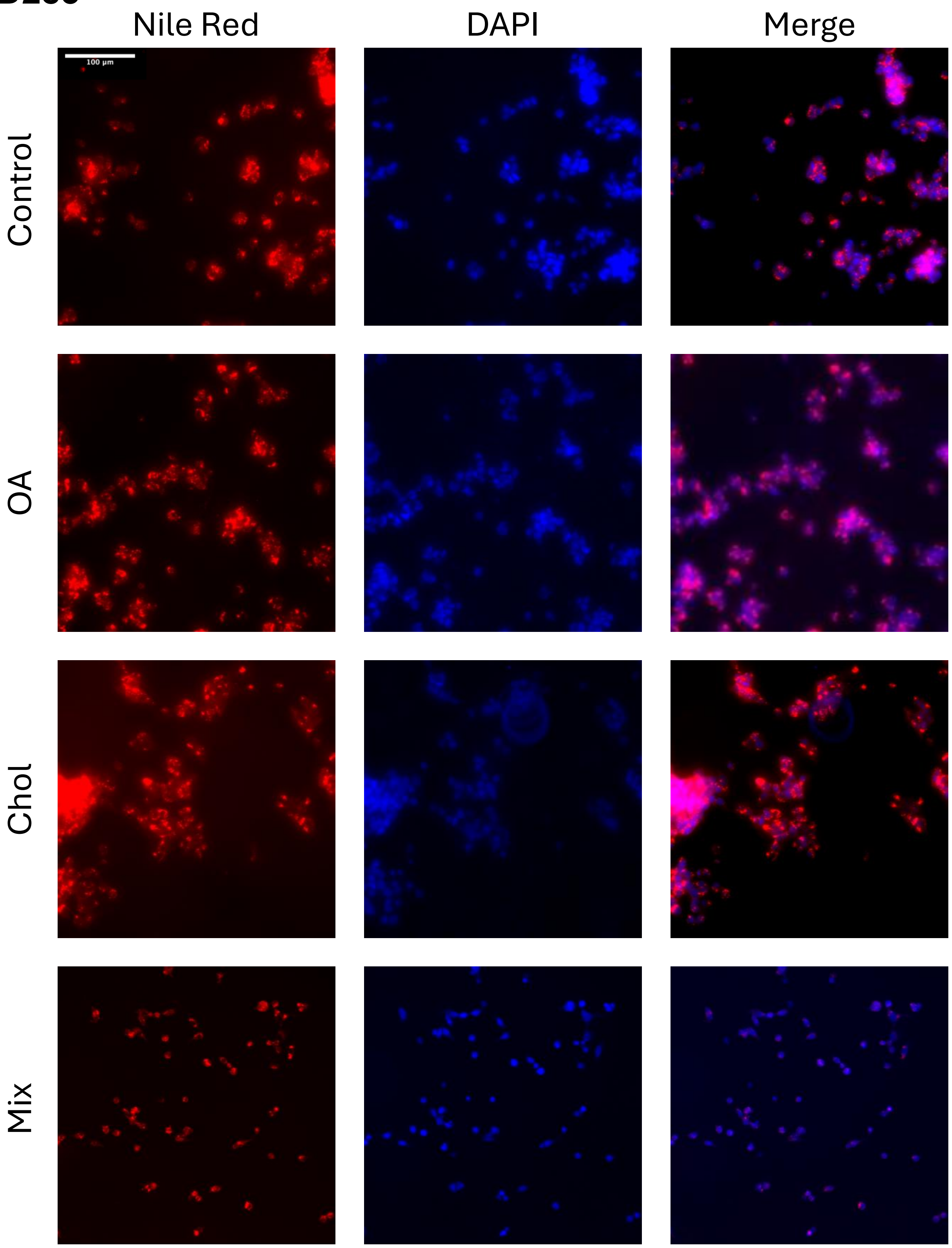

Supplement: Supplementary file 1 [file Data_Sheet_1.PDF]
